# Personalized Communication with Patients at the Emergency Department—An Experimental Design Study

**DOI:** 10.3390/jpm12101542

**Published:** 2022-09-20

**Authors:** Gillie Gabay, Attila Gere, Glenn Zemel, Howard Moskowitz

**Affiliations:** 1 Achva Academic College, Arugot 7980400, Israel; 2 Institute of Food Science and Technology, Department of Postharvest, Supply Chain, Commerce and Sensory Science, Hungarian University of Agriculture and Life Sciences, 1118 Budapest, Hungary; 3 Dupage Valley Anesthesiologists, Inc., Naperville, IL 60101, USA; 4 Mind-Genomics Associates, White Plains, White Plains, NY 10617, USA

**Keywords:** clinicians, communication, emergency department, experimental design, mindsets, patient satisfaction, regression

## Abstract

Communication of clinicians at the emergency department is a barrier to patient satisfaction due to lack of human connection, lack of control over the situation, low health literacy, deficient information, poor support at a time of uncertainty all affecting perceived quality of care. This explorative study tests drivers of patient satisfaction with communication of clinicians at the emergency department. The sample comprises 112 Americans from the New York greater area, who visited an emergency department in the past year. A conjoint-based experimental design was performed testing six messages in six categories. The categories encompass acknowledged aspects of communication with health providers enabling to compare among them when exploring communication at the ED by patient preferences. Respondents rated messages by the extent to which it drives their satisfaction with communication of clinicians at the emergency department. Based on the similarity of patients’ response patterns to each message, three significantly distinct mindsets of patient preferences regarding communication exchanges with clinicians at the emergency department emerged. Different conduct and communication messages drive the satisfaction of members of each mindset with the communication of clinicians at the emergency department. The strong performing messages for one mindset are irrelevant for members of other mindsets. Clinicians may identify the patient-belonging to a mindset and communicate using mindset-tailored messages. This novel strategy may enable clinicians to implement patient-centered communication, by mindset, promoting patient satisfaction and enabling clinicians to better cope with patients in the chaotic emergency department environment.

## 1. Introduction

Patients arriving to the ED experience a sudden change in their routine perhaps evoking anxiety and fear [1]. Pay-for-performance health plans monitor patient satisfaction making the subjective measure of patient satisfaction an increasingly important component of value-based quality of care [2]. Patient satisfaction is associated with benefits of patient collaboration, cooperation, adherence and improved clinical outcomes [3,4,5]. Satisfaction scores of patients affect the clinicians’ reimbursement and the hospitals’ financial incentives [1,6].

Communication with patients is an overarching category of patient satisfaction in the ED. Positive interpersonal exchanges in communication encourages questions, enhance internal locus of control and health literacy, create a human connection, demonstrate respect in non-verbal language, confidential, honest, compassionate, reliable allowing patient involvement [3,7,8,9,10]. Negative interpersonal exchanges in communication entail a bad bed side manner, lack of listening, provoking anxiety, condescending and lacking empathy, creating negative feelings and experiences of objectification, all negatively affecting perceived quality of care [11,12,13].

Poor interpersonal exchanges between patients and clinicians at the ED result in a gap in quality of care increasing length of hospitalization and costs, decreasing the efficient utilization of resources [14]. Additionally, poor communication is associated with frustration and dissatisfaction of patients with ED care [1,15]. Research also indicates that a third of the complaints regarding visits to the ED involved poor communication of clinicians with patients, regardless of age, gender, education, and status [16,17]. Poor communication in the ED is associated with lack of human connection, lack of control over the situation, low health literacy, deficient information, and poor support at a time of uncertainty [18]. Dissatisfaction with communication of clinicians negatively impacts perceived quality of care and may increase the risk for adverse health outcomes [19,20,21]. Thus, communication quality impacts patient outcomes [6]. Poor communication remains a significant barrier to optimal patient satisfaction with visits to the ED [1,20].

Overcoming the communication barrier at the ED will lead to better patient understanding, trust, satisfaction, and improved outcomes [22]. Effective communication alleviates the frustration patients experience due to long waiting times [23]. Thus, meeting patient expectations regarding communication exchanges with clinicians at the ED is essential to patient satisfaction across countries [1,20]. It is essential to consider the patients’ perspectives regarding drivers of satisfaction with communication of clinicians that may focus on personal, emotional, and psychological needs in addition to clinical care [24]. Although clinicians spend more than two thirds of the patient-encounter time in intrapersonal communication, only a minority of them focus on patient centered care entailing personal, emotional, and psychological needs of patients [22,25]. Patient-centered communication improves both patient experience and provider wellness [26]. Despite much evidence regarding the centrality of communication to patient experience, health outcomes, and clinicians wellness, communication is an insufficient organizational strategy [27].

Known drivers of patient satisfaction with communication of clinicians at the ED are: Interpersonal skills (expressive quality, information delivery, responsiveness, availability) [28]; comfort and bedside manner (perceived friendliness, courtesy, respect and compassion) [18,19,20,28,29]; encouragement to ask questions and use of jargon-free language [30]; empathy [31,32]; sufficient information that clinicians provide patients [4,33] and clarity about the role of different clinicians [1].

Despite the knowledge on these drivers of patient satisfaction with communication, to date, their implementation in communication at the ED is challenging and unsuccessful [3,34,35]. This study seeks to better understand patient preferences regarding communication with clinicians in the ED and identify specific communication messages that accord to patient preferences, as a necessary step in promoting patient satisfaction with communication of clinicians in the ED [16]. Studies on specific communication messages that clinicians may use when communicating with patients at the ED to promote their satisfaction, are scant [4,19]. This study focuses on patients’ perspectives regarding drivers of satisfaction with communication exchanges with clinicians at the ED, focusing on personal, emotional, and psychological needs in addition to clinical care. Responding to calls of previous studies, this study seeks to start to fill the gap in the state of the art by testing specific messages that ED clinicians may use in their interactions with patients to promote patient satisfaction with communication of clinicians in the ED [1,3,16,36,37]. The research question is: “Which communication messages drive patient satisfaction with the communication of clinicians in the ED?” The answer to this question may enable ED clinicians to select fewer yet more effective communication messages to improve patient satisfaction with communication in the ED.

## 2. Materials and Methods

### 2.1. Ethics

Upon participation in this online study participants signed an informed-consent form for both participation and publication. Participants were informed that they could stop their participation in this online study at any time.

### 2.2. Sample and Setting

The sample comprised 112 American respondents, ages 18–80 with 58 females and 54 males from the New York greater area. This sample size is consistent with the suggested sample size in conjoint analysis studies, particularly when aiming at stability of coefficients rather than stability of means and standard deviations [38,39,40]. Inclusion criteria were people 18 and over who visited the ED in a tertiary hospital at least once in the past year. Visits to the ED in a tertiary ED are memorable and people relate to them with specificity [3,41]. However, we limited the range of time so memories from the visit are still fresh on one hand and there is a potential of exploring variances by visit frequency, on the other hand. Respondents were not incentivized and represent a cross-section of the typical patient at the ED. Table 1 presents the demographic profile of the sample. Luc.id, Inc., a panel provider, invited respondents who met the inclusion criteria to participate, incentivized them and rerouted them to an online study on communication in the ED.

### 2.3. Procedure

We utilized an experimental design requiring the allocating participants to different groups using repeated measures, where the same participants took part in each condition of each of the independent variables (within groups, or within-subjects design) [42]. Thus, participants rated a series of different combinations of messages with the same rating question. Participants did not rate “parallel measures” but were repeatedly exposed to the same question, in relation to different aspects of communication with clinicians at the ED [42]. The experimental design enables, compared to typical observational studies, higher variation, randomization, analysis of co-variance and control reducing biases [42]. Since our reality is complex, encompassing many stimuli that may interact with one another, we utilized a conjoint-based experimental design well acknowledged in both academia and industry for uncovering the power of messages in a great variety of topics [43,44]. We applied the conjoint-based experimental design to uncover the power of specific messages as drivers of patient satisfaction with communication exchanges with clinicians in the ED. Numerous messages were tested (4032) with no limitation of degrees of freedom [45]. Each respondent evaluated a unique set of 48 combinations of messages, created by the basic experimental design [38]. With 112 respondents, each rating 48 combinations, this study covered 4032 messages.

### 2.4. Instrument

As typical in conjoint-analysis messages fall into categories each with messages. The dependent variable was ‘satisfaction with clinician-patient communication at the ED’. The independent variables were six categories of acknowledged communication elements of patient-centered care that drive patient satisfaction with communication of clinicians [46,47]. Categories were empathy, comfort, and bedside manner; providing sufficient information to patients; interpersonal skills of clinicians; encouragement to ask questions, avoiding use of jargon-free language and presenting the different roles and responsibilities of different clinicians [28,29,30,31,32,33]. Each category contained six messages, strictly one from each category, altogether thirty-six different messages. Messages were created based on elements we identified in a thorough literature search regarding independent variables and on previously published studies on patient expectations from communication of clinicians [3,48,49].

The categories of messages contained one message from each category. Participants were instructed to rate the combination as a unity [39,45]. The rating question was: “To what extent does the following combination of messages drive your satisfaction with clinicians’ communication in the ED?” The rating question appeared on each screen above the combination of messages. This outcome variable was rated on a scale ranging from 1 (Does not at all drive my satisfaction) to 9 (Strongly drives my satisfaction). The order of the combinations of messages was dictated by the well-crafted mathematical method underlying the experimental design, which structured the 48 combinations to ensure statistical independence of the predictor variables for subsequent regression at both the group level and the individual level [38,39,45]. Table 2 presents the study instrument.

### 2.5. Data Analysis

The experimental design enabled the deconstruction of responses to the messages by ordinary least-squares regression (OLS) [39,45,50]. With 112 respondents each rating 48 vignettes, we created 4032 models for satisfaction with communication were created using OLS, one for each respondent, each with both an additive constant and 48 coefficients, one coefficient for each message. The additive constant is a purely estimated parameter, the intercept in a linear equation that may be interpreted as the predisposition of the respondent group to agree to a set of messages in the absence of any specific message. High additive constants (60+) represent groups of people who are likely assign a high rating to the presented vignettes. Messages with low values, or negative values, detract from the high level of ratings. Low additive constants (<35) represent groups of people who are likely to assign low ratings to the presented vignettes. In such cases the specific messages drive satisfaction.

We performed OLS to generate individual level equations for each respondent relating to the presence/absence of the thirty-six messages. The OLS coefficient is the conditional probability that the specific message adds to the satisfaction. A coefficient of six or higher is statistically significant, given the standard error of about 4 for the coefficient. A higher coefficient means higher satisfaction. OLS was run for the total panel and for each key subgroups (gender, age), incorporating all relevant data into one regression model for the sample. The response to these vignettes, uncovered by OLS, reveals the part-worth contribution of each message to satisfaction with judgment bias reduced [45]. Since the self-ratings of respondents are not calibrated, following OLS we transformed the rating to a categorical variable (1–6 = 0; 7–9 = 1) enabling reduction of variability and crystallization of the strongest drivers of satisfaction with communication of clinicians at the ED.

Next, we analyzed response patterns to each message, using k-means clustering algorithm with 1-Pearsons’s R distance measure [51]. Fundamental groups, ‘mindsets’, emerged. ANOVA and Post Hoc tests indicated that the mindset models were significant. These mindsets highlight the different specifics of communication that drive satisfaction, for members of each mindset. The pattern of positive high coefficients across different subgroups guided the assignment of respondents to mindsets [51]. Last, to translate the knowledge derived in this study to practice, we developed a prediction tool, the personal viewpoint identifier (PVI). The PVI tool is a web-based tool by which clinicians may quickly assign a person waiting at the ED to a mindset in the sample. The PVI is based on converting six of the strongest distinguishing messages to binary questions (agree or disagree) that the patient rates. The six messages were chosen using a Monte-Carlo simulation [52]. Each of the 216 possible patterns of responses to the set of six messages is best associated with one of the three mindsets. Based on answers to the six binary questions in the PVI, the individual is assigned to one of the mindsets [44].

## 3. Results

### 3.1. Descriptive Analysis

The response rate for the on-line sample was a high response rate of 82%. To test the reliability, we created three sets of coefficients: from the total panel, and from each half-set. The two half sets of data were highly correlated with data for the total panel (0.90 for group 1; 0.87 for group 2).

### 3.2. Secondary Analysis

We created models for satisfaction with communication of clinicians using OLS, one model for each respondent, each with an additive constant and 48 coefficients (i.e., one coefficient for each message). The additive constant is an estimated parameter representing the intercept in a linear equation that may be interpreted as the predisposition of the respondent group to agree to a set of messages in the absence of any specific message. The response to each combination of messages, the coefficient of the OLS, reveals the power that each respondent attributes to each message as a driver of satisfaction [45].

To highlight the best-performing messages and eliminate a high variability due to lack of calibration among respondents, we transformed the ratings to a binary scale. Ratings 7,8, and 9 (upper 33% of the scale) were transformed to 100, classified as powerful drivers and ratings below 6 (lower 66% of the scale) were transformed to 0, classified as weak or negative drivers. OLS analysis was performed to create an individual-level regression model for each respondent. This type of individual regression approach has been widely used in conjoint analysis studies [39,45]. The OLS model was written as follows: $\hat{Y}=\beta_{0}+\beta_{1}X_{1}+\beta_{2}X_{2}+\cdots +\beta_{p}X_{p}$, where $\hat{Y}$ is the predicted or expected value of satisfaction (here, the transformed, binarized ratings), $X_{1}$ through $X_{p}$ are p distinct independent or predictor variables, $\beta_{0}$ is the value of Y when all of the independent variables ($X_{1}$ through $X_{p}$) are equal to zero, and $\beta_{1}$ through $\beta_{p}$ are the estimated regression coefficients. The OLS coefficient is the conditional probability that the specific message adds to additive constant for the satisfaction with communication. OLS was run for the entire panel, incorporating all relevant data into one regression model for the sample. The regression model, estimated at the level of each respondent, is appropriate because of the permuted design.

To simplify the analysis, we present only messages with positive regression coefficients, driving satisfaction with communication of clinicians in the ED. Negative regression coefficients mean either that the message is neutral (irrelevant for satisfaction) or counterproductive, driving dissatisfaction. Regression coefficients for the models relate to the presence/absence of the messages to the rating of drives/does not drive, after binary transformation. (*denotes significant, positive model parameters (*p* < 0.05)). Table 3 shows that as far as the estimation of ED Communication Messaging Models for the Total Sample, the additive constant is extremely high (85), suggesting that respondents view the topic of satisfaction with communication of clinicians in the ED as important. The t and *p* values of the OLS regression in Table 3 indicate that coefficients of all messages, are not significant. There are no specific messages that drive patient satisfaction with communication of clinicians at the ED. While respondents may react differently to messages in communication of clinicians in the ED, there was no variability by gender, by age or by the number of visits in the ED. Table 3 presents analysis results.

### 3.3. Estimation of ED Communication Model for Subgroups and PVI

K-means clustering was applied on the 48 coefficients to create clusters [51]. Three mindsets merged from the commonality in response patterns to each message [52]. Following mathematical clustering, the equation for each subgroup was estimated using all data from the appropriate group [52]. One-way analysis of variance coupled with Tukey post hoc test indicates that differences among the mindsets, representing distinct models of communication, are significant, highlighting the different messages that drive satisfaction with communication for members of each mindset. The pattern of positive high coefficients across different mindsets guided the assignment of respondents to mindsets. The data suggest three distinct groups, emerging from the k-means clustering. Patients belonging to mindset 1 seek an acknowledgement that they are experiencing a crisis, mostly by listening to them. Patients belonging to mindset 2 seek information and physical privacy. Patients belonging to mindset 3 seek empathy and anxiety alleviation. The dominant messages that drive satisfaction in each mindset characterize it. These three mindsets transcend age, gender, and visit frequency. Table 4 presents the additive constant, coefficients for the specific messages that patients rated as strongly driving their satisfaction with communication of clinicians in the ED for each mindset, while superscript letters indicate the results of Tukey post hoc test. Bold messages in Table 4 are messages with significant coefficients (*p* < 0.05) that emerged from k-means clustering.

Since the three mindsets are distributed across the population, a PVI is required to identify the belonging of individuals in the population to a mindset in the sample.

## 4. Discussion

This study tested the power of numerous communication messages as drivers of patient satisfaction with communication of clinicians in the ED. This study makes several contributions. Theoretically, clustering by the similarity in patients’ patterns of response to messages is a novel strategy which revealed three distinct mindsets, similar in size, representing what drives patient satisfaction with communication of clinicians in the ED for members of each mindset. Methodologically, this study used a patented methodology of conjoint-based experimental design, overcoming the typical biases of surveys, and simultaneously testing various messages crafted to reflect the complexity in our reality which affects patient’s satisfaction with communication of clinicians at the ED. Practically, the web-based prediction tool enables clinicians to quickly identify the mindset-belonging of each patient and communicate with each patient using mindset-tailored messaging.

The novelty of this explorative study is a breakthrough in removing barriers to patient satisfaction with communication of clinicians by identifying the mindset-belonging and using mindset-tailored specific messages. Findings indicate that patients have different response patterns to different communication messages of clinicians. In contrast to prior studies that viewed patient satisfaction as inflfluenced by patient sociocultural, psychosocial, and disease-related characteristics [33], our findings, suggest that the traditional segmentation by ‘who people are’ is insufficient for an in-depth understanding of drivers of patient satisfaction with communication of clinicians at the ED. The mindsets suggest that using the same messages for all patients does not promote their satisfaction. Ineffective messages fall under the category of ‘what patients should know (e.g., “we are here for you”; “waiting times are long”) rather than reflect the experience of the patient (“acknowledgment of crisis”, understanding patients’ emotional needs”) [37]. This finding echoes previous studies claiming that that content-oriented messages do not promote satisfaction, compared to process-oriented messages that were found to be a ‘make or break’ in patient satisfaction [7].

To promote patient satisfaction, communication should be accorded to mindset-belonging. Patients who belong to mindset 1, need clinicians to carefully listen to them, acknowledge them as individuals and enable them to feel comfortable. This finding supports previous findings on the importance of acknowledging the patient visiting the ED as experiencing a time of uncertainty, perhaps a crisis, and communication should aim at enhancing patient wellbeing [4,5,8]. Patients who belong to mindset 2, need information and explanations about their illness and the treatment process. They also need privacy regarding personal information. Patients who belong to mindset 3, need clinicians to alleviate their anxiety, clarify their role and responsibility, allow family or friends to sit with the patient, and respect patients’ physical privacy.

These findings echo a recent study on the four voices of clinicians: a content task-oriented voice, a process-oriented voice, a comprehension-oriented voice and a learning orientation amongst which clinicians need to shift [6]. While in the latter study the shift is situation-dependent, the mindsets are patient tailored aiming at promoting satisfaction and improved outcomes. Messages that were strong drivers of satisfaction were: “The clinician carefully listened, showed interest in me as a person”; “From the start I knew what was the role of the clinician in my room”; and “Clinicians attended to pain control.” This finding supports studies on the association between the perceived intention of clinicians, their caring behaviors, and patient satisfaction.

Using communication by mindsets, clinicians may improve patient satisfaction regardless of the setting, the diagnosis, or the demographics. The strategy of using a few targeted mindset-tailored messages, may facilitate patient-centered communication even among ED clinicians working at a chaotic work environment, who find such communication as challenging [37,53]. Further education may be required. The web-based prediction tool assigning patients into a mindset will allow clinicians to identify the mindset-belonging of the patient at the ED and communicate targeted mindset-tailored messages to promote patient satisfaction extending other apps that improve patient experience at the emergency department [54]. Future studies may explore the fit of messages as culturally grounded for shaping communication with patients by distinct chronic illnesses and replicate this research with the PVI tool developed in this explorative study.

Since respondent are people who accepted the invitation to participate in this study, they may carry a self-selected bias. Additionally, the geographic area from which respondents were recruited, may limit the generalization of this study to other countries. Last, it is also possible that the association between satisfaction and communication messaging is affected by other aspects of respondents’ visits to the ED.

## 5. Conclusions

This study starts to fill a knowledge gap in the state of the art examining targeted communication messaging as means to raise patient satisfaction with communication of clinicians in the ED. Understanding the drivers of satisfaction regarding communication of clinicians in the ED is essential to patient satisfaction. Patients, however, have different preferences of communication. Establishing communication by mindsets in a practical and functional manner may carry implications for delivery of care on the local, national, and international level. Thus, the knowledge derived from this explorative study highlights a potential novel approach and a new communication tool of mindset-tailored messaging. The use of the PVI, employs technology as a useful tool for improving communication.2 ED clinicians may identify patients by their belonging to one of three mindsets and use communication messages by patient mindset-belonging, highlighting a few messages with each patient throughout the visit to the ED. Although encounters between vulnerable patients and clinicians in the ED are short and fragmented, we present a potential path to overcome the communication barrier and promote satisfaction. Tailoring communication by mindsets may bridge the gap between bio-clinical care and psycho-social care [15]. Clinicians are called upon to use the PVI by asking the right few questions, identifying the belonging of each patient to a mindset in the sample, and use the appropriate communication messages with members of each Mindsets in their visits to the ED.

To promote patient satisfaction with communication of clinicians in the ED, clinicians may identify patient-belonging to a mindset and communicate using mindset-tailored messages. These results highlight a novel strategy enabling clinicians to implement patient-centered communication, by mindset-belonging, in the delivery of care. Since the work environments in emergency departments is chaotic, clinicians may use only a few messages that are effective drivers of patient satisfaction for members of each mindset, to better cope with the complexity of patient encounters in the emergency department.

## Figures and Tables

**Table 1:** Demographics of the Sample.

Total Sample (*n*)		112
Gender	Male	48%
	Female	52%
Age	18–29	19%
	30–39	18%
	40–49	17%
	50–59	16%
	60–69	23%
	70–79	6%
	80+	1%
Race	White	81%
	Black/African American	9%
	Hispanic	5%
	Native American	1%
	Asian	4%
	Other	1%
Marital status	Married	47%
	Divorced	21%
	Never Married	27%
	Widow/Widower	5%
Family income	<$20,000	16%
	$20,000 to $49,000	31%
	$50,000 to $99,000	38%
	$100,000 to $149,000	9%
	$150,000 to $199,000	4%
	>$200,000	2%
What is the highest degree or level of school you have completed?	Less than high school	4%
	High school or equivalent	28%
	Some college	21%
	Associate Degree	13%
	Bachelor’s degree	24%
	Graduate Degree	11%
How many people in your household including yourself?	1	30%
	2	33%
	3	10%
	4	16%
	5	6%
	6 or more	5%
What is the setting of where you live?	City/Urban	35%
	Suburban	40%
	Rural	25%
Approximately how many visits to an Emergency Department have you had in the past year?	1–2	49%
	3–4	27%
	5–6	14%
	>6	10%

**Table 2:** Six Categories and Six Messages in Each comprising the Instrument.

	**Category A: Empathy**
A1	Clinicians care about me as a person
A2	Clinicians are concerned about my comfort
A3	Clinicians ask about my condition
A4	Clinicians are courteous when they take my information
A5	Clinicians monitor my condition
A6	Clinicians are empathic
	**Category B: Provide Information**
B1	Clinicians keep me informed
B2	Clinicians are clear
B3	Clinicians explain things to me
B4	Clinicians keep my family informed
B5	Clinicians keep me informed about delays
B6	Clinicians provide me with concise written discharge instructions
	**Category C: Interpersonal skills**
C1	Clinicians carefully listen to me
C2	Clinicians addresses my needs
C3	Clinicians are discreet...respect my privacy
C4	Clinicians show interest in me as a person
C5	Clinicians pay attention to pain control
C6	Clinicians respond patiently and promptly
	**Category D: Comfort**
D1	Clinicians make efforts to minimize my wait time
D2	Clinicians allow family and friends to sit with me
D3	Clinicians move me through the process as quickly as possible
D4	Clinicians move me quickly to the treatment area
D5	Clinicians assure that I am comfortable in the waiting area
D6	Clinicians see me quickly after my arrival
	**Category E: Encouraging questions, Avoiding Jargon and Role Clarity**
E1	Clinicians reframe from using medical jargon
E2	Clinicians maintain a calm and quiet setting
E3	Even from the start...I always know the role of the clinician in my room
E4	Clinicians treat me gently during exam
E5	Clinicians are experienced making me comfortable about procedures
E6	Clinicians encourage me to ask questions
	**Category F: Bedside Manner**
F1	Clinicians are courteous to my family and friends
F2	Clinicians are attentive even in cases of long waiting times
F3	Clinicians address my physical AND mental states
F4	Clinicians guarantee privacy of my personal information
F5	Clinicians are compassionate
F6	Clinicians are there to help me

**Table 3:** The Power of all 36 Messages as Drivers of Patient Satisfaction in the Total Panel.

Message by Descending Part Worth Contribution	Coefficient	Standard Error	T Statistic	*p*-Value
Additive constant	85	4.0	21	0.0
Experienced clinicians so you are comfortable	2.5	1.9	1.3	0.2
Clinicians reframe from using medical jargon	2.3	1.9	1.2	0.2
Clinicians ask about your condition	2.3	1.9	1.2	0.2
Clinicians see me quickly after my arrival	2.2	1.9	1.1	0.3
Clinicians explain things to me	2.1	1.0	1.1	0.3
Clinicians guarantee privacy of personal information	2.0	1.0	1.0	0.3
Clinicians treat me gently during exam	1.9	1.2	1.0	0.3
Clinicians are concerned about my comfort	1.3	2.0	0.6	0.5
Clinicians assure I am comfortable	1.1	2.0	0.6	0.6
Clinicians are compassionate	1.1	2.0	0.6	0.6
Clinicians allow family and friends to sit with me	1.1	1.9	0.6	0.6
Clinicians move me quickly to the treatment area	1.0	1.9	0.5	0.6
Clinicians are clear	0.9	2.0	0.5	0.6
Clinicians care about me as a person	0.9	2.0	0.5	0.7
Clinicians make efforts to minimize my wait	0.6	1.9	0.3	0.8
Clinicians are empathic	0.6	2.0	0.3	0.8
Clinicians keep me informed about delays	0.5	2.0	0.2	0.8
Clinicians carefully listen...show interest in me	0.4	2.0	0.2	0.8
Clinicians maintain a calm and quiet setting	0.1	2.0	0.1	1.0
Clinicians encourage me to ask questions	−0.3	1.9	−0.2	0.9
Clinicians are helpful	−0.3	2.0	−0.2	0.9
Clinicians are clear	−0.4	2.0	−0.2	0.9
Clinician addresses both my physical & mental states	−0.4	2.0	−0.2	0.8
Clinicians keep me informed	−0.5	2.0	−0.3	0.8
Clinicians address my needs	−0.5	2.0	−0.3	0.8
Family and friends are kept informed of the care plan	−0.7	2.0	−0.3	0.7
Clinicians are courteous	−0.8	2.0	−0.4	0.7
Even from the start... I always know the role of the clinician in my room	−1.1	2.0	−0.5	0.6
Clinicians carefully attend to pain control	−1.1	2.0	−0.5	0.6
I know clinicians are there to help me	−1.4	2.0	−0.7	0.5
Clinicians are courteous with my family and friends	−1.5	1.9	−0.8	0.4
Clinicians are discreet...respect my privacy	−1.5	2.0	−0.8	0.4
Clinicians provide me with clear and concise written discharge instructions	−1.6	2.0	−0.8	0.4
Clinicians are courteous when taking my personal information	−2.1	2.0	−1.1	0.3
Clinicians respond patiently and promptly	−2.7	2.0	−1.4	0.2
Clinicians are attentive even in cases of long waiting times	−3.2	2.0	−1.6	0.1

**Table 4:** Mindset-Segmentation (MS) for Drivers of Satisfaction with Clinician-Patient communication in the Emergency Department and Post hoc ANOVA test.

	MS1	MS2	MS3
Base size	38	38	36
Additive constant	44	56	29
Segment 1—They pay attention to me and make me feel comfortable			
Clinicians carefully listen...show interest in me as a person	**8**	0	5
Clinicians allow family and friends to sit with me	**6**	−7	−1
Segment 2—They control my pain and respect my privacy			
Clinicians carefully attend to pain control	0	0	**8**
Clinicians are discreet...respect my privacy	−1	−2	**7**
Segment 3—They know what they are doing, and are professional about it			
Clinicians explain things to me	2	**6**	−3
Even from the start... I always know the role of the clinician in my room	−7	**9**	0
Clinicians are compassionate	1	**6**	−4
Clinicians keep me informed	2	**6**	−4
Clinicians are concerned about my comfort	4	**6**	−6

In bold, significant coefficients as emerged from K-means clustering (*p* < 0.05).

## Data Availability

The data presented in this study are available on request from the corresponding author.
